# In Situ Stabilisation of Silver Nanoparticles at Chitosan-Functionalised Graphene Oxide for Reduction of 2,4-Dinitrophenol in Water

**DOI:** 10.3390/polym13213800

**Published:** 2021-11-03

**Authors:** Rebaone Makaudi, Hugues Kamdem Paumo, Boniface Kamdem Pone, Lebogang Katata-Seru

**Affiliations:** 1 Department of Chemistry, Faculty of Natural and Agricultural Sciences, North-West University, Mmabatho 2735, South Africa; rebaonemakaudi@gmail.com; 2 Department of Pharmacy, Faculty of Pharmaceutical Sciences, University of Sao Paulo, Sao Paulo 05508-900, Brazil; ponekamdemboniface@gmail.com

**Keywords:** 2,4-dinitrophenol, supported Ag nanoparticles, graphene oxide, chitosan, catalytic transformation

## Abstract

This investigation reports the in situ growth of silver nanoparticles onto covalently bonded graphene oxide-chitosan, which serve as supported nanocatalysts for the NaBH_4_ reduction of 2,4-dinitrophenol in aqueous systems. Fumaryl chloride reacted with chitosan in an acidic environment to yield a tailored polymeric material. The latter was, in turn, treated with the pre-synthesised graphene oxide sheets under acidic conditions to generate the GO-functionalised membrane (GO-FL-CS). The adsorption of Ag^+^ from aqueous media by GO-FL-CS yielded a set of membranes that were decorated with silver nanoparticles (Ag NPs@GO-FL-CS) without any reducing agent. Various analytical tools were used to characterise these composites, including Fourier transform infrared spectroscopy, Brunauer-Emmett-Teller surface area analysis, X-ray diffraction, scanning electron microscopy/energy-dispersive X-ray analysis, inductively coupled plasma-mass spectrometry, and transmission electron microscopy. The silver-loaded materials were further used for the remediation of 2,4-dinitrophenol from aqueous solutions under batch operation. The BET analysis revealed that the functionalisation of GO with chitosan and Ag NPs (average size 20–60 nm) resulted in a three-fold increased surface area. The optimised catalyst (Ag mass loading 16.95%) displayed remarkable activity with an apparent pseudo-first-order rate constant of 13.5 × 10^−3^ min^−1^. The cyclic voltammetry experiment was conducted to determine the nitro-conversion pathway. The reusability/stability test showed no significant reduction efficiency of this metal-laden composite over six cycles. Findings from the study revealed that Ag NPs@GO-FL-CS could be employed as a low-cost and recyclable catalyst to convert toxic nitroaromatics in wastewater.

## 1. Introduction

Freshwater ecosystems represent only 3% of the earth’s surface water and is distributed throughout rivers, lakes, and dams [1,2]. These are considered to be the main sources of clean water for humans and wildlife. However, the fast-growing population, increased agricultural activity and expanding chemical industry have put a strain on the quality of this ecosystem. The contamination of freshwater bodies has seen a notable rise throughout the past two decades due to technological innovations and industrialisation. The pace of industrialisation has proceeded far beyond the capability of regulators to implement meaningful restrictions on the level of point source pollution that is acceptable to the various industrial complexes [3]. The discharge of industrial wastewater into the environment has caused severe damage to the quality of the freshwater ecosystem. Treatment of these effluents represents one of the feasible approaches that can reduce the contamination of bodies of water. Amongst various pollutants identified in wastewater, nitroaromatics have been described as exhibiting acute toxicity to aquatic organisms and human health. These compounds are very stable in an aqueous environment and have demonstrated estrogenicity and mutagenicity [4]. This characteristic property is ascribed to the –NO_2_ group interacting with the reductase enzymes and generating the reactive oxygen species [5]. Interestingly, this group can also undergo hydrogenation in proton and electron donors to yield the less toxic aminoaromatics.

Several technologies have been reported to reduce nitroarenes’ levels in aqueous media, including the solid-catalyst-assisted hydrogenation process [6]. The supported noble metal nanocatalysts make a noteworthy contribution to the heterogeneous catalysis that transforms noxious organic compounds in aqueous solutions [7,8]. Recently, carbon-based supports have gained much interest for wastewater treatment, owing to their attributes such as low corrosion capability, high conductivity, high thermal stability, and relatively lower price [9]. Although the performance of noble metal nanocatalysts is satisfactory in terms of their synergistic action with the support matrix, high cost and limited availability are their major downsides [10,11].

Graphene oxide (GO) is a carbon-based material that is widely reported in wastewater remediation [12]. This is ascribed to its unique electronic property and the presence of surface oxygen functional groups, namely, carboxyl, hydroxyl, and epoxide. These polar groups account for the ability of GO to adsorb metal ions in an aqueous solution. Moreover, its conductivity is responsible for the commendable behaviour it exhibits in electron-transfer chemical development [13]. GO material allows for quick electron transport in redox reactions. However, the poor mechanical strength, hydrophilicity, and stable dispersion of this material pose considerable setbacks. The oxygen-containing groups of GO can also serve as active seed points for the condensation of GO with a polymeric material. The covalent functionalisation of GO with organic compounds is a popular technique for improving its limitations [14,15].

The functionalisation of GO with a polymer that bears the surface amino groups may facilitate the nucleation, deposition, and immobilisation of noble metal nanoparticles onto the support structure [16]. Three main strategies have been developed in the literature for the preparation of supported nanocatalysts. The first method involves the pre-fabrication of metal nanocatalysts followed by immobilisation onto a solid support using a stabilising agent [17]. The second strategy entails the in situ nucleation, growth, and immobilisation of the metal nanocatalysts onto support structures in the presence of an external reductant [18]. Recently, the in situ nucleation, growth and stabilisation of Ag nanocatalysts along the magnetic mercaptoacetic acid-functionalised polypyrrole (Fe_3_O_4_@PPy-MAA) support material was reported to proceed without the aid of a reducing agent and stabiliser [19]. This development was attributed to electron-rich thiol and amino groups at the surface of the functionalised conducting polymer PPy.

In the current investigation, we sought to develop a relatively low-cost material that could support in situ nucleation, growth, and stabilisation of Ag nanoparticles in adsorption procedures, with no reductant and stabiliser. This study describes the simple preparation of a four-component, GO-fumaryl chloride-chitosan-Ag NPs (GO-FL-CS-Ag NPs) hybrid nanomaterial. In the first step, the pre-fabricated GO sheets are crosslinked with a bio-derived and inexpensive chitosan polymer through ester bonding using fumaryl chloride. The following step involves the reduction between silver ions and the amino-rich GO-FL-CS, and the subsequent formation of Ag NPs at room temperature under organic solvent-free conditions. The kinetic investigation of the conversion of the harmful 2,4-dinitrophenol in water, using the developed Ag NPs that were deposited onto the GO-FL-CS membrane, is presented. The GO-FL-CS material and Ag-adsorbed derivatives were characterised by FTIR, SEM-EDX, TEM, BET, XRD, and ICP-MS techniques. The catalytic reduction of 2,4-DNP using the Ag NP-decorated GO-FL-CS was demonstrated, and the reaction route was proposed using voltammetric measurements. The reusability of the optimised catalyst was also tested.

## 2. Materials and Methods

### 2.1. Materials

Graphite flakes, sulfuric acid (H_2_SO_4_ 98%), sodium nitrate (NaNO_3_), potassium permanganate (KMnO_4_), hydrochloric acid (HCl 37%), hydrogen peroxide (H_2_O_2_ 30%), thionyl chloride (SOCl_2_), fumaric acid, pyridine, chitosan (MW 50–190 kDa with deacetylation degree > 90%), chlorosulfonic acid (CSA), sodium borohydride (NaBH_4_), ammonium hydroxide (NH_4_OH) and silver nitrate (AgNO_3_) were procured from Sigma-Aldrich, Johannesburg, South Africa. These reagents were used as obtained. All aqueous solutions were prepared using ultrapure water. Acronyms and parameters used in this article are listed in Table 1.

### 2.2. Preparation of GO Sheets

The GO sheets were prepared according to the Hummers’ method as reported elsewhere, with some modifications [20]. Graphite flakes (5 g) and NaNO_3_ (2.5 g) were added to 115 mL of H_2_SO_4_ under stirring for an hour. Subsequently, KMnO_4_ (15 g) was slowly introduced while cold-holding the reaction mixture in an ice bath. The combination was then vigorously stirred at 35 °C overnight and diluted with 2.5 L of ultrapure water. The resulting suspension was isolated and re-dispersed in 25 mL of H_2_O_2_. The as-prepared GO sheets were treated with HCl, washed with water, and freeze-dried.

### 2.3. Preparation of Fumaryl Chloride (FL)

The process of chlorinating fumaric acid was carried out using the protocol described elsewhere [21,22]. A solution of FL was prepared by dissolving fumaric acid (5 g) in 40 mL of thionyl chloride, followed by the dropwise addition of 4 mL pyridine. Next, the reaction mixture was vigorously stirred under reflux overnight and allowed to cool to room temperature. The excess of SOCl_2_ was removed by evaporation at 50 °C under reduced pressure.

### 2.4. Preparation of FL-CS

The tailored polymeric material FL-CS was prepared following a method reported by Wu et al. [23] with few amendments. Briefly, chitosan powder (2.5 g) was dissolved in 50 mL of CSA at room temperature. The resulting solution was homogenised for 10 min, and 4 mL of pyridine was inserted. The as-prepared solution was then added slowly to the solution of fumaryl chloride under stirring in an ice bath. The reaction mixture was stirred for 6 h. The precipitate formed was isolated by filtration, neutralised using a solution of NH_4_OH, washed thoroughly with water, and freeze-dried.

### 2.5. Preparation of GO-FL-CS Membrane

A mixture of the prepared GO sheets (0.8 g) and FL-CS (0.8 g) was stirred in 50 mL of CSA in an ice bath for 10 min. Subsequently, 2 mL of pyridine was added to the solution, and stirring was continued overnight. Ice-cold water was then added to the reaction, and the resulting precipitate was isolated by filtration and treated as described for the synthesis of FL-CS.

### 2.6. Preparation of GO-FL-CS Membrane Decorated with Ag NPs (Ag NPs@GO-FL-CS)

The prepared GO-FL-CS membrane was used as an adsorbent for the fabrication of Ag NPs@GO-FL-CS. In a typical batch adsorption operation, an aqueous solution of AgNO_3_ (100 mg/L) was prepared, and a GO-FL-CS composite (0.2 g) was added to 500 mL of this solution. The system was kept under stirring (500 rpm) at room temperature for 5 h. Thereafter, the silver-loaded membrane was isolated, washed with ultrapure water, air-dried at room temperature for 48 h, and labelled as Ag NPs@GO-FL-CS 100. This operation was repeated using solutions of 200 and 300 mg/L aqueous silver ions, and the obtained silver-loaded membranes were designated as Ag NPs@GO-FL-CS 200 and Ag NPs@GO-FL-CS 300, respectively.

### 2.7. Characterisation

The structural information of the prepared GO, GO-FL-CS, and Ag NPs@GO-FL-CS materials was investigated by XRD patterns using a Bruker D8 advance diffractometer (Bruker Optics, Berlin, Germany) with a Cu-Kα radiation monochromatic filter (λ = 0.154 Å). The morphology and nanostructural information of the samples were studied using a JEOL-JSM 7500F SEM microscope (JEOL, MA, USA) coupled with an EDX accessory and a JEOL-JEM-1010 TEM microscope (JEOL, Tokyo, Japan) operating at an accelerating voltage of 100 kV. The particle-size distribution was determined by counting and analysing the particles with ImageJ. The surface functional groups of CS, GO, FL-CS, GO-FL-CS, and the Ag-loaded membrane were examined by FTIR spectroscopy using a Bruker Alpha FTIR-ATR spectrometer (Bruker Optics, Ettlingen, Germany). The BET surface area of GO, the GO-functionalised membrane and silver-decorated derivatives were measured using a NOVA touch LX^2^ Quantachrome instrument (Quantachrome, FL, USA). The silver-loading onto GO-FL-CS was determined using an Agilent 7500 series ICP-MS spectrometer (Agilent, Santa Clara, CA, USA). The samples were digested in HNO_3_ (70%) at 160 °C and 60 bar pressure for 30 min, and analysed using a Total Quant method. A spectrophotometer PerkinElmer Lambda 365 (PerkinElmer, Waltham, MA, USA) was employed to evaluate the concentration of 2,4-DNP in the reaction environment during the Ag NP-catalysed transformation.

### 2.8. Catalytic Reduction Experiments

The catalytic reduction study was conducted using synthetic wastewater that contained 2,4-DNP (1.23 mM, pH 4.67) as a representative organic contaminant. This solution was prepared by dissolving 2,4-DNP (0.231 g) in 1 L of water. In addition, a solution of NaBH_4_ (0.25 M) was prepared to serve as an H-donor for the catalytic H-transfer process.

A solid catalyst weighing 2.5 mg was added to 25 mL of the 2,4-DNP solution under stirring (500 rpm) at 25 °C. Subsequently, 2.5 mL of a freshly prepared H-donor solution was added to the mixture. The NaBH_4_ reduction of 2,4-DNP at the solid catalyst was monitored through the change in absorbance values over time. The reduction rate was evaluated using the Langmuir-Hinshelwood kinetic expression simplified to the pseudo-first-order kinetics [24] as represented by

ln([2,4-DNP]_t_/[2,4-DNP]_0_) = −kt

where t, [2,4-DNP]_0_, [2,4-DNP]_t_, and k denote the contact time (min), initial concentration (mM) of 2,4-DNP, the concentration of 2,4-DNP at a given time t, and apparent rate constant (min^−1^), respectively. The value of k was computed from the slope of the plot of ln(A_t_/A_0_) versus t. A represents the absorbance value. The effect of the silver-loadings was studied by assessing the absorbance value of 2,4-DNP and the apparent reaction rate constant in the presence of the GO-FL-CS membrane and the Ag-loaded derivatives (Ag NPs@GO-FL-CS 100, Ag NPs@GO-FL-CS 200, and Ag NPs@GO-FL-CS 300). The dependence of the 2,4-DNP reduction at the optimised Ag-loaded membrane was also conducted using various dosages (2.5, 5, and 10 mg) while keeping other key parameters (pH, 2,4-DNP concentration, NaBH_4_ concentration, temperature, and stirring speed) as constant.

The best dose was further used to assess the stability and reusability of the optimised nanocatalyst in successive cycles of reduction under the same reaction conditions. After each cycle, the catalyst was air-dried at room temperature for 48 h. The electrochemical measurements were performed using a glass–carbon electrode to suggest a rational reduction route. For this experiment, the sample solution of 2,4-DNP in contact with Ag NPs@GO-FL-CS was prepared in a buffer (0.1 M acetate buffer solution at pH 4.5).

## 3. Results and Discussion

### 3.1. Synthesis of Ag NPs@GO-FL-CS

**Scheme 1:**
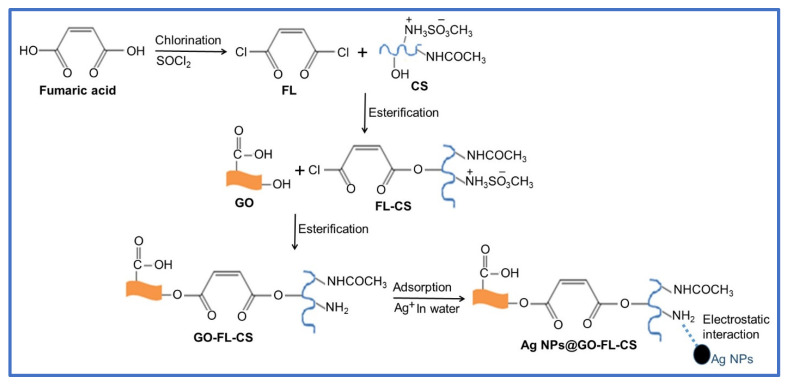
Schematic diagram for the fabrication of Ag NPs@GO-FL-CS.

The attachment of fumaryl chloride with a biodegradable CS polymer led to the formation of FL-CS, a polymeric material that could provide multifunctional sites for chemical bonding with GO sheets and physical bonding with metal ions. The reaction pathways that were followed to synthesise GO-FL-CS, which was used as a membrane for the growth and immobilisation of Ag NPs, are outlined in Scheme 1. The highly reactive FL linker was prepared by base-promoted chlorination of fumaric acid with thionyl chloride. Pyridine was used as a base to prevent the hydrolysis of the sulfonate intermediate. This dihalogenated compound was subjected to nucleophilic substitution with CS in acidic conditions. The reaction proceeded through the protonation of the amino groups of CS and substitution onto the –OH functionalities. Accordingly, the GO sheets were synthesised using the modified Hummers’ method and covalently functionalised with the prepared FL-CS polymer in acidic treatment. This reaction took place via esterification of the GO hydroxyl groups. The as-prepared GO-FL-CS membrane was allowed to interact with the silver ions in aqueous media to generate the Ag NPs@GO-FL-CS composites. The electron-rich –NH_2_ groups on the membrane acted as reductant and stabilising sites for the growth and immobilisation of Ag NPs.

### 3.2. Characterisation

**Figure 1:**
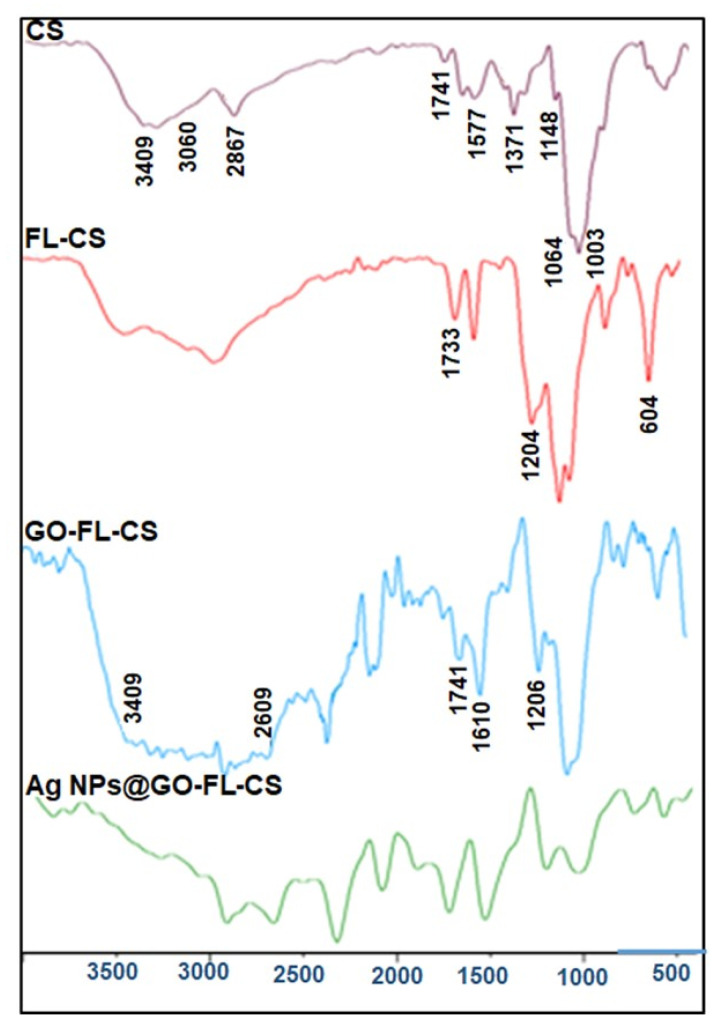
FTIR spectra of CS, FL-CS, GO-FL-CS, and Ag NPs@GO-FL-CS.

Figure 1 represents the FTIR spectra of CS, FL-CS, GO, GO-FL-CS, and Ag NPs@GO-FL-CS. The characteristic bands of CS due to –OH and –NH stretching vibrations were recorded in the region 3400–3000 cm^−1^. The bands at 2867, 1741, 1645, 1577, 1420, 1371, 1148, 1064, and 1023 cm^−1^ were due to C–H asymmetric stretching, C=O deformation, C=O stretching of primary amide, N–H bending of primary amine, CH_2_ symmetrical deformation, CH_3_ symmetrical deformation, C–O–C bridge asymmetric stretching, C–O stretching in secondary alcohols and C–O stretching in primary alcohols, respectively. These data are consistent with those of other investigations [25,26]. From the spectrum of FL-CS, the covalent bonding from the nucleophilic substitution between the alcoholic –OH of CS and the –COCl of FL was assumed. The modified CS polymer exhibited a relatively broad intensity of the bands corresponding to the hydroxyl contents. Moreover, a sharp absorption band was observed at 604 cm^−1^. This was ascribed to the C–Cl frequency [27]. Intense bands were also observed at wavenumbers of 1733 and 1204 cm^−1^, corresponding to C=O and C–O stretching vibrations of the ester. The band intensity of the C–O stretching in primary alcohols showed a substantial decline [28].

The preparation of GO-FL-CS was assessed by comparing its FTIR results with that of FL-CS. The GO-FL-CS spectrum exhibited a relatively strong and broad band in the region 3500–2300 cm^−1^ due to increasing alcoholic –OH levels and –OH stretching of carboxylic acids arising from GO. The distinctive absorption bands at 1741 and 1206 cm^−1^ were attributed to carbonyl stretching of the ester group and C–O epoxy stretching, respectively. The increased band at around 1610 cm^−1^ was due to C=C functional groups. However, no absorption bands corresponding to amino-functionalised GO were observed [23,29]. Furthermore, the reduced intensity of the band corresponding to C–Cl showed that this group participated in the covalent attachment of GO. These results indicated that the GO sheets were functionalised with FL-CS through the hydroxyl groups. Upon treatment with Ag^+^ ions in an aqueous solution, the GO-FL-CS membrane was characterised by the reduced intensity of the band corresponding to N–H/O–H stretching. This suggested that these functional groups acted as binding sites for Ag^+^ uptake, certainly by chelation. Functional groups such as –SH, –SO_3_H, –NH_2_, –OH, and –COOH have been found to demonstrate a strong binding affinity for heavy metal ions [19]. Remarkably, these species have high standard reduction potential and can easily undergo reduction to the corresponding zero-valent metal during the sorption process.

**Figure 2:**
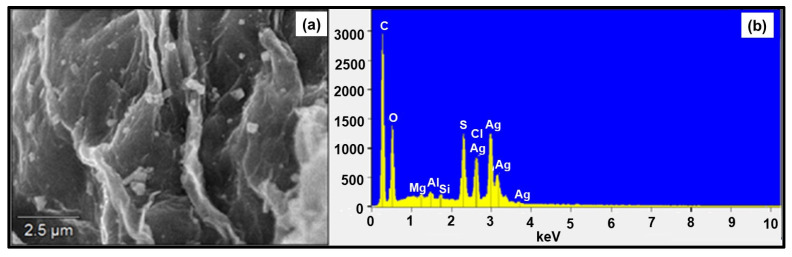
(**a**) SEM micrograph of Ag NPs@GO-FL-CS 100 and (**b**) the EDX analysis spectrum.

The SEM image of GO-FL-CS material treated with Ag^+^ ions displayed a membrane with a rough surface and distribution of granules (Figure 2a). This result suggested the accumulation of metal ions onto the GO-functionalised membrane and the development of metallic nanocrystals. A distribution of the silver that had adsorbed on the membrane surface was confirmed using the EDX characterisation technique (Figure 2b and Table 2). Peaks corresponding to C, O, S, Cl, and Ag elements were detected at 0.277, 0.525, 2.307, 2.621, and 2.984 keV, respectively. The appearance of prominent Ag peaks reflected the chelating ability of the prepared GO-FL-CS material. The existing S and Cl peaks were attributed to the presence of residues of sulphur-containing compounds during the preparation of GO-FL-CS, and seemingly the occurrence of AgCl. The ICP-MS investigation revealed that the silver mass that was loaded onto the GO-FL-CS surface was 4.06, 16.95, and 11.91 wt% after treatment in 100, 200, and 300 mg/L aqueous silver ions solutions, respectively.

**Figure 3:**
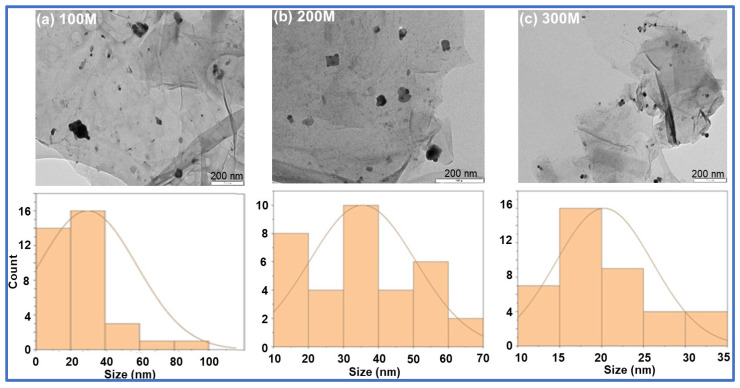
TEM images of (**a**) the Ag NPs@GO-FL-CS 100, (**b**) Ag NPs@GO-FL-CS 200, and (**c**) Ag NPs@GO-FL-CS 300 composites and the corresponding Ag particle size distribution histograms.

**Figure 4:**
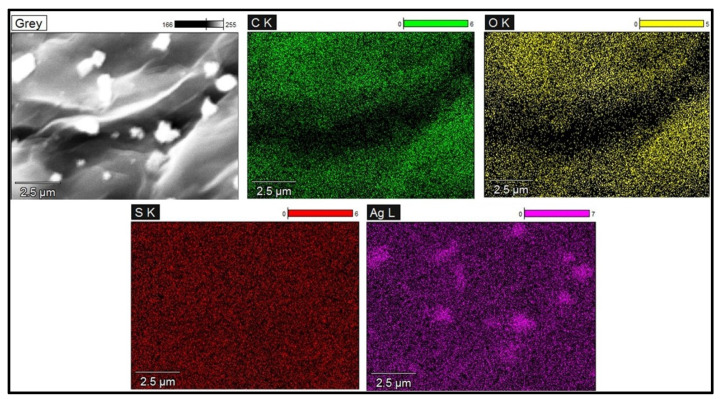
STEM elemental mapping of Ag NPs@GO-FL-CS 300.

The TEM analysis displayed the nanoparticles distributed on the surface of the GO-FL-CS matrix. The size distribution of these particles ranged from 20 to 60 nm. The Ag NP-decorated GO-FL-CS membranes that were developed using silver nitrate solutions of 100, 200, and 300 mg/L revealed Ag crystallites with average sizes of 30.0, 35.5, and 20.3 nm, respectively (Figure 3). The increase in concentration of silver ions to 300 mg/L led to the decrement of particle size. This effect is ascribed to the limited number of surface amino groups available for the silver-reduction process [30]. A relatively lower metal precursor concentration (200 mg/L), on the other hand, resulted in broad grain size distribution. This is presumably due to the increased disorder at the interface of the GO-FL-CS adsorbent/Ag^+^ solution [31]. STEM elemental mapping was executed to assess the distribution of these particles on the surface of the GO-FL-CS adsorbent. Figure 4 shows a uniform dispersion of C (green), O (yellow), and S (red) throughout the membrane surface after adsorption. The STEM as well as TEM analysis illustrated that the Ag NPs were randomly spread throughout the adsorbent matrix.

**Figure 5:**
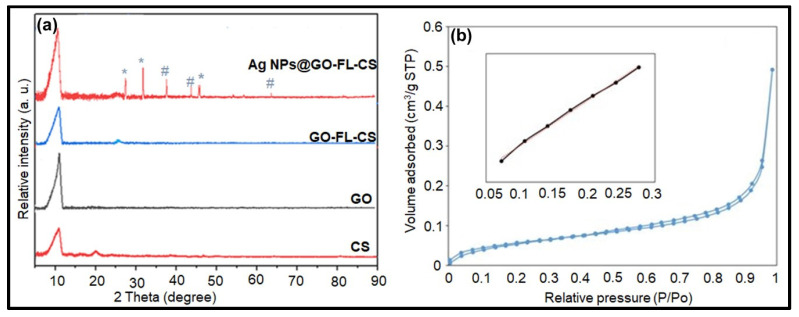
(**a**) XRD patterns of GO, CS, GO-FL-CS, and Ag NPs@GO-FL-CS; Peaks attributed to crystalline phase of (*) AgCl and (#) Ag NPs; (**b**) N_2_ adsorption-desorption isotherms of Ag NPs@GO-FL-CS.

XRD analysis was also used to obtain additional structural information of the GO-FL-CS membrane and the Ag NP-trapped derivatives. Figure 5a shows the XRD patterns of CS, GO, GO-FL-CS, and Ag NPs@GO-FL-CS. The diffractogram pattern of GO revealed a broad peak at the Bragg angle (2θ) 10.69°, which is characteristic of the *d*-spacing of 0.83 nm [32,33], while the CS pattern consisted of two broad peaks at 2θ 10.81° and 19.86° due to the presence of intra- and intermolecular hydrogen bonds between the CS chains [34]. The GO-FL-CS diffraction spectrum showed a broad peak at 2θ 10.94°, which was attributed to the polymeric FL-CS material, and a band at 2θ 26.20° that was assigned to the covalently functionalised GO with an interlayer distance of 0.31 nm. This result is consistent with a previous study on the modification of GO [35]. The decrease in *d*-spacing suggests a loss of oxygen functional groups and a functionalisation of the –OH along the edges [29]. Furthermore, it can be observed that the GO-FL-CS membrane, after the sorption of Ag^+^ ions, exhibited three characteristic sharp diffraction peaks at 2θ 38.15, 44.41, and 64.43°, which correspond to the (111), (200), and (220) crystal planes of Ag, respectively [36]. This result serves as evidence for the conversion of adsorbed Ag^+^ ions to Ag^0^ at the GO-FL-CS network. Previously, it has been reported that the presence of a reducing reagent is fundamental for the nucleation of Ag and/or Au NPs at the functionalised GO-CS surface [37]. This GO-based material was prepared by the N-substitution of CS. The peaks that were attributed to a highly crystalline phase of AgCl were also observed at 27.68 (111), 32.35 (200), and 46.29° (220). However, the low weight % of Cl indicated that AgCl NPs were in very low amounts.

The surface area of the prepared materials was examined by conducting the N_2_ adsorption-desorption analysis. The isotherms for GO, GO-FL-CS, and Ag NPs@GO-FL-CS showed the same general shape, that is, the type H3 hysteresis loop (Figure 5b). This is indicative of the porous nature of the prepared composites. The specific surface areas were determined to be 26.04, 40.40, and 62.99 m^2^/g for GO, GO-FL-CS 100, and Ag NPs@GO-FL-CS 200, respectively. The surface area was increased by three-fold following the covalent functionalisation of GO with FL-CS and the immobilisation of Ag nanocrystals on the resulting GO-FL-CS support. The heterogeneous catalytic reactions are surface interactions. An increase in the specific surface area of supported nanocatalysts is likely to improve their activity.

### 3.3. Catalytic Reduction of 2,4-DNP

Aminophenolic compounds are useful ingredients in several applications such as cosmetics, photographic development, coating-lubricant additives, and antipyretic and analgesic drugs [38]. Herein, we used the reduction of 2,4-DNP to aminophenol with the H-donor of NaBH_4_ as a model system to quantitatively assess the catalytic efficiency of the prepared Ag NPs@GO-FL-CS composites.

**Figure 6:**
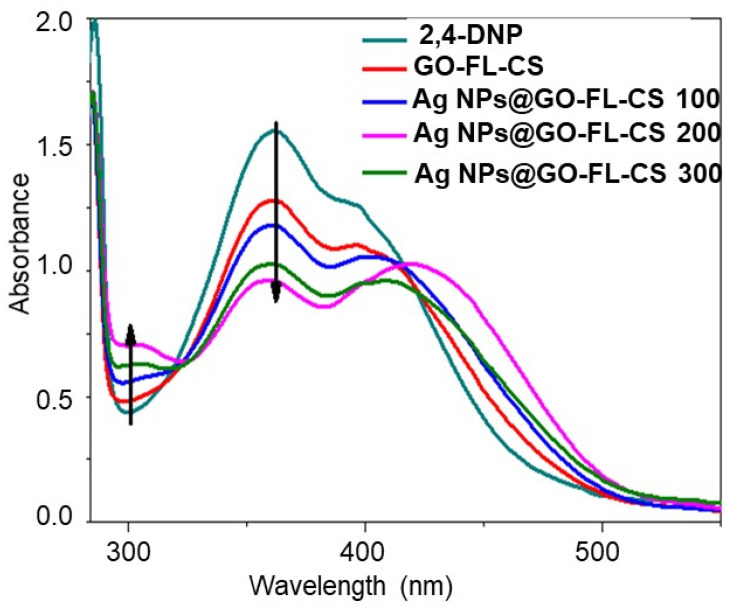
UV-Vis spectra for the reduction 2,4-DNP in 30 min treatment time.

The aqueous solution of 2,4-DNP displayed a strong absorption band at 359 nm and a shoulder at a lower frequency of 394 nm. These bands are ascribed to the intramolecular charge transfer character, involving the excitation of the π-electrons of the phenol structure’s highest-occupied energy level to a vacant energy level of the –NO_2_ groups [39]. The catalytic transformation of 2,4-DNP with NaBH_4_ in excess was evaluated by a change in the absorbance of the major band and a bathochromic shift to 448 nm, in accordance with the previous literature [40]. Before quantitatively evaluating the catalytic activity of Ag NP-loaded GO-FL-CS composites, the role of the GO-FL-CS support during the reduction of 2,4-DNP was tested. Figure 6 shows a decrease in the concentration of 2,4-DNP for the NaBH_4_-assisted reduction in the presence of pristine GO-FL-CS after 30 min of contact time. A further increase in contact time to 60 min did not show significant changes. This indicated that the GO-FL-CS membrane is capable of binding 2,4-dinitrophenolate ions through π-stacking [41]. It has been demonstrated that the hydrogenation of nitrophenol after NaBH_4_ hydrolysis is also kinetically limited with no catalyst [42,43]. The latter serves to limit the repelling phenomenon between the occurring borate ions (BO_2_^–^) and phenolate species. The development of Ag NPs onto the GO-FL-CS adsorbent led to the significant consumption of 2,4-DNP in the aquatic media, as compared to pristine GO-FL-CS. Moreover, Ag NPs@GO-FL-CS 200 exhibited the lowest concentration of 2,4-DNP, as compared to Ag NPs@GO-FL-CS 100 and Ag NPs@GO-FL-CS 300.

#### 3.3.1. Optimisation of Ag Loading

**Figure 7:**
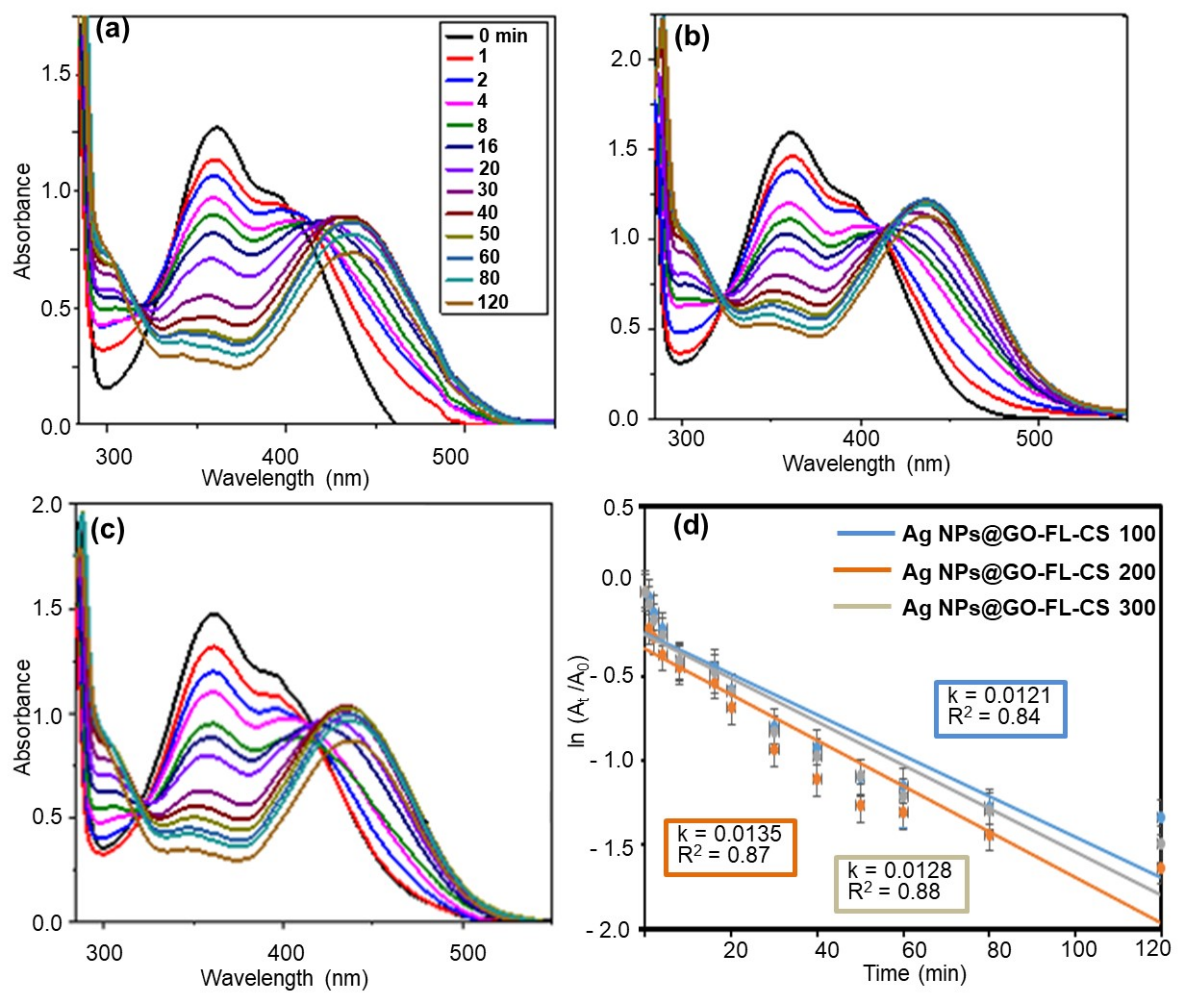
UV-Vis absorption spectra for the reduction of 2,4-DNP over (**a**) Ag NPs@GO-FL-CS 100, (**b**) Ag NPs@GO-FL-CS 200, and (**c**) Ag NPs@GO-FL-CS 300; (**d**) The corresponding kinetics graphs. The error bars denote standard deviations obtained from three trials.

A comparative study of the 2,4-DNP reduction kinetics of Ag NPs@GO-FL-CS was performed (Figure 7a–d). The apparent reduction rates were found to be (1.21 × 10^−2^) ± (4.39 × 10^−4^), (1.35 × 10^−2^) ± (4.57 × 10^−4^), and (1.28 × 10^−2^) ± (2.01 × 10^−4^) min^−1^ for a solution in contact with Ag NPs@GO-FL-CS 100, Ag NPs@GO-FL-CS 200, and Ag NPs@GO-FL-CS 300, respectively. This shows that Ag NPs@GO-FL-CS 200 performed relatively better. The results obtained in this experiment are in agreement with the EDX and ICP-MS data that showed a higher Ag loading for Ag NPs@GO-FL-CS 200. Nevertheless, this composite exhibited Ag NPs with larger sizes (10–70 nm) than Ag NPs@GO-FL-CS 300 (10–35 nm). Consequently, the increasing reduction rate of Ag NPs@GO-FL-CS 200 could also be attributed to the presence of a significant number of grains that were larger than 50 nm. These have been reported to display a higher number of active sites at the vicinity of the particle boundaries [44]. Considering the results obtained by ICP-MS analysis and the reduction kinetics of Ag NPs@GO-FL-CS, the optimised concentration of Ag at the surface of GO-FL-CS was 16.95 wt%.

#### 3.3.2. Influence of Ag NPs@GO-FL-CS Dosage

**Figure 8:**
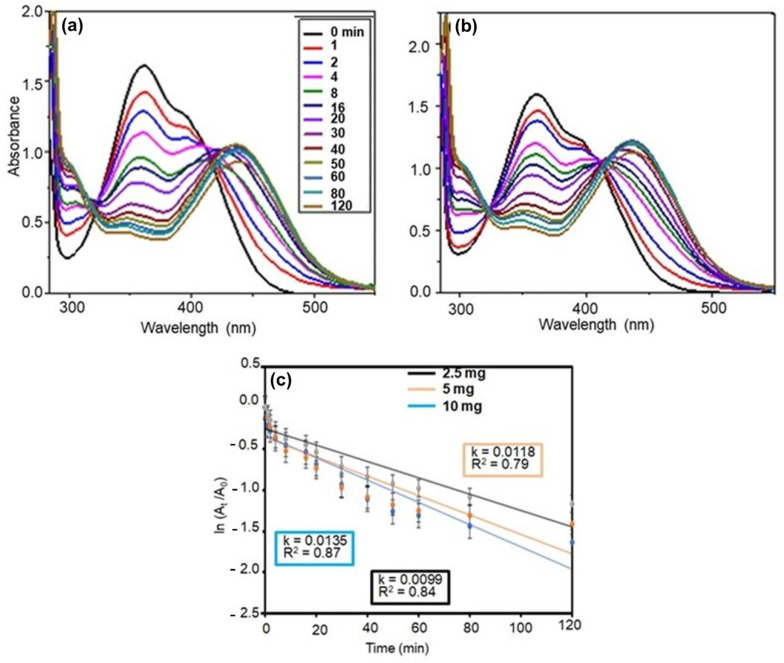
UV-Vis absorption spectra for the reduction of 2,4-DNP over Ag NPs@GO-FL-CS 200 using (**a**) 5 mg and (**b**) 10 mg; (**c**) The corresponding kinetics graphs.

The dependence of the 2,4-DNP reduction on Ag NPs@GO-FL-CS was investigated using various dosages (2.5, 5, and 10 mg). Figure 8a–c present the reduction performance of Ag NPs@GO-FL-CS 200 in terms of dosage. The calculated rate constants were (1.18 × 10^−2^) ± (2.57 × 10^−4^), (1.35 × 10^−2^) ± (4.57 × 10^−4^), and (9.97 × 10^−3^) ± (2.18 × 10^−4^) min^−1^ using 2.5, 5, and 10 mg doses, respectively. It can be observed that the magnitude of the reduction efficiency increases as the amount of Ag NPs@GO-FL-CS 200 increases from 2.5 to 5 mg. This is certainly due to an upsurge in the number of active catalytic sites as the concentration of Ag NPs increases. However, a further increase in the Ag NPs@GO-FL-CS-Ag 200 dose to 10 mg resulted in a significant decline in the 2,4-DNP reduction rate. This may be attributed to the overlapping of binding and active catalytic sites. The adsorption of BH_4_^−^ and 2,4-DNP onto the surface of supported nanocatalysts is fundamental for the hydrogen-transfer reaction to proceed [45].

#### 3.3.3. Mechanism of Reduction

**Figure 9:**
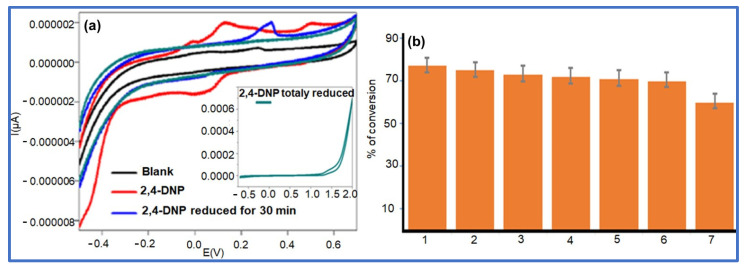
(**a**) Cyclic voltammetry curves obtained with solutions of 2,4-DNP before and after treatment with Ag NPs@GO-FL-CS; (**b**) Reusability test.

**Scheme 2:**
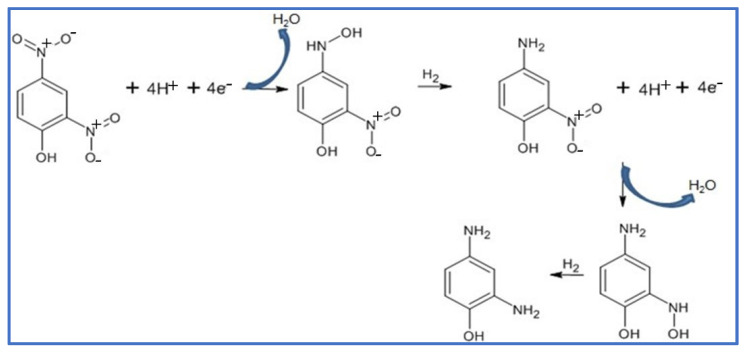
Proposed mechanism for the BH_4_^−^ reduction of 2,4-DNP using Ag NPs@GO-FL-CS nanocatalyst.

The NaBH_4_ reduction of 2,4-DNP in aqueous media using an Ag NPs@GO-FL-CS supported nanocatalyst was proposed to progress through the adsorption and hydrolysis of BH_4_^−^ ions to generate the silver hydride complex. At the same time, the adsorption of 2,4-dinitrophenolate was also anticipated to take place. The co-adsorption process was then followed by the transfer hydrogenation of the adsorbed dinitrophenolate species [46]. However, this molecule exhibits two competing –NO_2_ groups. The voltammetric measurements of the solutions of 2,4-DNP before and after treatment with Ag NPs@GO-FL-CS 200 were carried out in order to elucidate the conversion path. This electrochemical technique is a popular and highly sensitive method for measuring the redox property of a compound. Hence, this technology can provide qualitative information about the activity of an Ag NPs@GO-FL-CS composite by determining the generated species in an aqueous solution after treatment. The sample solution, which was obtained after a 30 min treatment, showed a disappearance of the peaks that correspond to the presence of the 2,4-DNP analyte (Figure 9a). Moreover, the emergence of a well-defined reduction peak at 0.33 V was attributed to the reduction of a –NO_2_ group. In accordance with previous studies [47,48], this suggests the reduction of the –NO_2_ at the *para*-position. The relatively sterically hindered –NO_2_ group at the *ortho*-position is foreseen to undergo reduction with difficulty. Previously, it has been demonstrated that the relaying number of electrons and protons is the same during the reduction of *para*-nitrophenol and *ortho*-nitrophenol [49]. The findings concluded that the conversion of 2,4-DNP at Ag NPs@GO-FL-CS, in the presence of the H-donor BH_4_^–^, proceeded through an irreversible sequential four-electron/-proton transfer at the *para*- and *ortho*-positions (Scheme 2).

#### 3.3.4. Reusability of the Ag NPs@GO-FL-CS Nanocatalyst

The reusability experiment of the Ag NPs@GO-FL-CS supported nanocatalyst was carried out through several consecutive reduction runs of 2,4-DNP in the presence of an NaBH_4_ reductant using 5 mg of Ag NPs@GO-FL-CS 200. This study is indispensable to the evaluation of the stability, cost-effectiveness, and potential applicability of a catalyst in large-scale water treatment. Monitoring the efficiency of Ag NPs@GO-FL-CS 200 through reuse cycles is a means to assess issues such as catalyst leaching and/or transformation while promoting 2,4-DNP conversion. Figure 9b shows the percentage of conversion of 2,4-DNP after a sixth consecutive use of Ag NPs@GO-FL-CS 200 (≈70%). This demonstrated no substantial loss of activity by the prepared nanocatalyst after six runs; hence, this system exhibited good chemical stability and low leaching. For this reason, the prepared GO-functionalised membrane (GO-FL-CS) was also considered as a good support for anchoring the Ag nanocatalyst.

## 4. Conclusions

The chitosan-functionalised graphene oxide containing the free NH_2_ groups was synthesised to serve as a support structure for the development of silver nanocatalysts without the aid of a reductant. These supported nanocatalysts were tested for the NaBH_4_ hydrogenation of environmentally toxic 2,4-DNP in an aqueous solution. FTIR spectra analysis showed that the fabricated GO was functionalised with FL-CS through ester bond formation, thereby leaving the electron-donating NH_2_ groups unreacted. A variety of characterisation techniques (SEM, EDS, TEM, STEM, and XRD) were utilised to demonstrate the strong affinity of the prepared GO-FL-CS membrane towards Ag^+^ ions. These techniques were also used to confirm the in situ nucleation, growth and stabilisation of Ag NPs with no reducing reagent. The ICP-MS data indicated that the Ag NPs@GO-FL-CS that was obtained from a 200 mg/L Ag^+^ ion solution exhibited the highest Ag mass loading (16.95%). This composite displayed a good catalytic efficiency for the reduction of 2,4-DNP, with the pseudo-first-order rate constant of 13.5 × 10^−3^ min ^−1^. The cyclic voltammetry study suggested that the reduction of 2,4-DNP progressed through the 4-amino-2-nitrophenol intermediate. The Ag NPs@GO-FL-CS material that was developed in this study could also be recovered and reapplied more than six times. Thus, this composite can serve as a cost-effective supported nanocatalyst to treat nitroaromatics in industrial effluents.

## Figures and Tables

**Table 1:** List of acronyms and parameters.

Ag NPs@GO-FL-CS	Silver Nanoparticles Stabilised at Graphene Oxide-Chitosan Membrane
CS	Chitosan
CSA	Chlorosulfonic acid
DNP	Dinitrophenol
FL	Fumaryl chloride
FL-CS	Covalently bonded fumaryl chloride and chitosan
GO	Graphene oxide
GO-FL-CS	Crosslinked graphene oxide-chitosan using fumaryl chloride
E	Potential (V)
I	Current (µA)
k	Apparent rate constants (min^−1^)
STP	Standard temperature and pressure

**Table 2:** Quantitative analysis of detected element using EDS mapping.

	Ag NPs@GO-FL-CS 100	Ag NPs@GO-FL-CS 200	Ag NPs@GO-FL-CS 300
Element	Weight %	Weight %	Weight %
C	51.96	48.49	49.61
O	22.98	11.19	22.09
S	10.32	7.88	5.55
Cl	3.29	4.60	Not detected
Ag	11.17	22.27	21.56
